# Decoding Brain Responses to Names and Voices across Different Vigilance States

**DOI:** 10.3390/s21103393

**Published:** 2021-05-13

**Authors:** Tomasz Wielek, Christine Blume, Malgorzata Wislowska, Renata del Giudice, Manuel Schabus

**Affiliations:** 1 Cognition and Consciousness Research, Laboratory for Sleep, Department of Psychology, University of Salzburg, Hellbrunner Strasse 34, 5020 Salzburg, Austria; wielekto@stud.sbg.ac.at (T.W.); Christine.Blume@upk.ch (C.B.); Malgorzata.Wislowska@sbg.ac.at (M.W.); 2 Transfaculty Research Platform Molecular and Cognitive Neurosciences, University of Basel, Birmannsgasse 8, CH-4055 Basel, Switzerland; 3 Centre for Chronobiology, Psychiatric Hospital of the University of Basel, Wilhelm-Klein-Str. 27, CH-4002 Basel, Switzerland; 4 Department of Health Sciences, Università Degli Studi di Milano, 20146 Milan, Italy; re.delgiudice@gmail.com

**Keywords:** sleep, EEG, decoding

## Abstract

Past research has demonstrated differential responses of the brain during sleep in response especially to variations in paralinguistic properties of auditory stimuli, suggesting they can still be processed “offline”. However, the nature of the underlying mechanisms remains unclear. Here, we therefore used multivariate pattern analyses to directly test the similarities in brain activity among different sleep stages (non-rapid eye movement stages N1-N3, as well as rapid-eye movement sleep REM, and wake). We varied stimulus salience by manipulating subjective (own vs. unfamiliar name) and paralinguistic (familiar vs. unfamiliar voice) salience in 16 healthy sleepers during an 8-h sleep opportunity. Paralinguistic salience (i.e., familiar vs. unfamiliar voice) was reliably decoded from EEG response patterns during both N2 and N3 sleep. Importantly, the classifiers trained on N2 and N3 data generalized to N3 and N2, respectively, suggesting similar processing mode in these states. Moreover, projecting the classifiers’ weights using a forward model revealed similar fronto-central topographical patterns in NREM stages N2 and N3. Finally, we found no generalization from wake to any sleep stage (and vice versa) suggesting that “processing modes” or the overall processing architecture with respect to relevant oscillations and/or networks substantially change from wake to sleep. However, the results point to a single and rather uniform NREM-specific mechanism that is involved in (auditory) salience detection during sleep.

## 1. Introduction

This research draws on recent findings by Blume et al. [1] who found that voice familiarity, that is, paralinguistic emotional stimulus aspects, are constantly evaluated during virtually all stages of human sleep. Specifically, during both NREM sleep (N1–N3) and REM sleep, there was a differential response in event-related potentials (ERPs) as well as oscillatory activity in the 1–15 Hz range to stimuli uttered by a familiar vs. unfamiliar voice. Particularly the unfamiliar voice triggered larger event-related synchronization in a broad frequency range as compared to a familiar voice, which suggests continued saliency during sleep [1]. The phenomenon that the sleeping brain preserves (at least to some degree) its ability to process sensory information has previously been shown for a range of stimuli including names [2,3], speech [4,5], complex auditory regularities [6], or arithmetic violations [7]. However, questions about the nature of the underlying mechanism continue to be a matter of debate. For instance, Blume and colleagues [1,3] suggest the existence of a coherent processing mode across all sleep stages (including REM)—a mode they call a ‘sentinel processing mode’. With respect to REM sleep, many previous studies either refrained from analyzing REM (for a review see [8]) or concluded that information processing in REM is altogether different from that observed during NREM [2].

Here, in order to test the hypothesis that there is one coherent processing mode across sleep stages, we re-analyzed the dataset previously published in [1] using multivariate decoding analyses. To this end, we included all stages from wake through NREM to REM sleep during a full night in the sleep laboratory. Multivariate decoding analyses aim to evaluate the overall underlying pattern of brain activity at the single trial level. Thus, it is considered more sensitive to effects that are distributed unevenly and variably across subjects than the classical analytical strategies such as ERP analyses [9]. Moreover, a decoder can be trained on one set of conditions (e.g., familiar vs. unfamiliar voice during wake) and tested on another (e.g., familiar vs. unfamiliar voice during REM), which provides a direct test of the similarities in brain activity between the two states, even in the presence of expected time delays in the neurophysiological response [10]. We also tested whether the decoder is able to generalize across different sleep stages, e.g., whether it can correctly classify stimuli in REM after being trained on N3 or a shallower sleep stage. Importantly, if generalization across states is possible, this supports the hypothesis of a single, coherent ‘sentinel processing mode’ [1,3] in sleep in contrast to clearly distinguishable processing modes between wake, light NREM, deep NREM and REM. We expected that wake patterns only generalize to N1 and, to a lesser extent and with a certain time delay, to light (N2) sleep, but not to deep (N3) or REM sleep. These hypotheses are based on the knowledge that N3 and REM represent quite different and distinct brain states with respect to the oscillatory composition of the EEG or connectivity [11] and of course (auditory) awakening thresholds [12]. Furthermore, it is increasingly well understood that these stages differ in the degree of residual information processing as shown for the tracking of speech [5], responsiveness to environmental stimuli such as word classification [13], or novelty detection [6,14].

## 2. Materials and Methods

### 2.1. Participants

Initially, 17 healthy participants (median 22.3 years, sd 2.3 years) were included in the analyses. Unfortunately, one participant did not reach REM sleep. In order to keep the number of participants constant across sleep stages, we decided to exclude that participant from all analyses.

### 2.2. Stimulation

**Figure 1:**
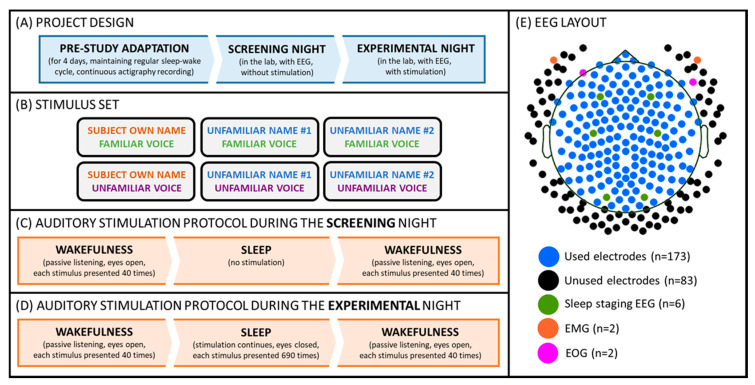
Experimental setup. (**A**) Participants kept a regular sleep-wake cycle for 4 days, and subsequently reported to the laboratory for screening and an experimental night. (**B**) Volunteers were stimulated with 6 different auditory cues presented in a pseudo-random order. (**C**) During the screening night, the stimulation was delivered during two “wakefulness” parts, which took place before and after the “sleep” part. (**D**) During the experimental night, the stimulation was delivered throughout two “wakefulness” as well as “sleep” parts. (**E**) The signal was recorded from 256 EEG channels during both (screening and experimental) nights. For sleep staging, two EMG (cf. orange dots), two EOG (cf. pink dots), and six scalp channels (i.e., F3/F4, C3/C4, O1/O2; cf. green dots) were used. For the final analyses presented here, a subset of 173 electrodes (cf. blue and green dots) was used.

Each participant came to the sleep laboratory of the University of Salzburg for an adaptation and an experimental night, during which s/he underwent a passive auditory stimulation procedure before and after sleep. Additionally, during the experimental night, the auditory stimulation continued during a regular 8 h sleep opportunity. During the stimulation, participants were presented with two types of names, that is, the subject’s own name vs. two unfamiliar names. Additionally, each of these names was uttered by two voices, namely a familiar voice (i.e., a parent or long-term partner) and an unfamiliar voice. During wakefulness, participants were asked to passively listen to the stimuli, which were presented at a volume of about 65 dB. During the experimental night, the volume was adjusted individually so that the stimuli were clearly audible, but participants could still sleep (cf. Figure 1). For more details please also see [1].

### 2.3. EEG Acquisition and Data Pre-Processing

Polysomnography was acquired with a high-density GSN HydroCel Geodesic Sensor Nets (Electrical Geodesics Inc., Eugene, OR, USA) in combination with a NetAmps 400 amplifier. Data were recorded at a sampling rate of 250Hz.

For the analyses, the initial 256 electrodes were reduced to 173. Specifically, we excluded the channels on the cheeks and in the neck due to muscle artefacts. The EEG signal was high-pass filtered at 0.5 Hz and an independent component analysis (ICA) based, semi-automatic artefact correction procedure (implemented in the Fieldtrip toolbox [15]) was conducted in order to remove eye and other non-neural artefacts (e.g., obvious ECG artefacts) from wakefulness data. The sleep data were pre-processed in a similar way, however without an exclusion of eye movements. Raw sleep data were scored by The Siesta Group© using Somnolyzer24x7 [16] according to standard AASM guidelines [17]. For further processing, data were segmented into 5 s epochs (from 2 s before to 3 s after stimulus onset), before a bandpass filter (1–20 Hz FIR filter) was applied. Then, we again segmented the data from 0.2 s before to 1 s after stimulus onset) thereby excluding potential filtering artifacts at the edges. Finally, we downsampled (by factor 4) to reduce the amount of data.

### 2.4. Decoding and Statistical Analyses

**Figure 2:**
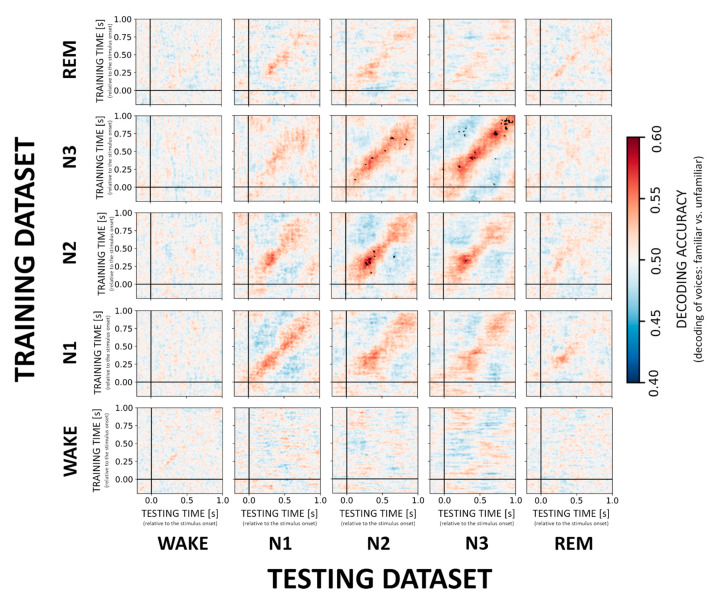
Cross-generalization matrices summarizing decoding performance for VOICE familiarity. The ability to discriminate between familiar vs. unfamiliar voices was evaluated within sleep stages (diagonal) and across sleep stages (off-diagonal). Color codes represent low (cold colors) to high (hot colors) classification accuracy (unit: percentage). Statistically significant results are highlighted with black dots. Note that VOICE stimuli can only be decoded in and between NREM sleep stages N2 and N3.

Multivariate classification started at −0.2 s and ended at 1.0 s relative to stimulus onset. In the first step, single-trial EEG data served as the input to the decoder or classifier, where the individual trials were low-pass filtered at 25 Hz and standardized by removing the mean and scaling to unit variance. Here, a classifier was trained to discriminate between two stimulus categories (e.g., familiar vs. unfamiliar voice) at every single time point using the information from the 173 EEG electrodes data. Training of the decoder was performed in MNE-Python using a temporal-generalization approach [9,18]. More specifically, during temporal-generalization it is evaluated whether a classifier, previously trained on a specific time point, can accurately classify any other time point of new “test epochs”. This results in cross-generalization matrices (e.g., Figure 2). 

For classification, a logistic regression with default regularization settings was used (L2 and C = 1) as recommended by Varoquaux and colleagues [19]. The classification performance was quantified using a standard accuracy score. Note that the number of epochs was balanced across sleep states by randomly sampling 100 epochs for each stimulus (except for N1, where all available epochs were used) from each condition to ensure that the chance level accuracy was kept at 50%. For more information on the sampling scheme including analyses with repeated sampling please see the supplementary material. To cross-validate (two-fold with stratification) the epochs, accuracy scores were computed for each subject. Significance was tested across subjects using a non-parametric (two-sided Mann-Whitney U) mass univariate analysis corrected for multiple comparisons with the Benjamini-Hochberg false discovery rate (FDR) procedure. To provide a topographical interpretation of the classifiers’ weights/coefficients, we used a solution implemented in MNE-Python which converts the raw weight vector (backward model) to a neurophysiologically interpretable forward model [20]. The goal of the backward model is to decode, that is, discriminate two stimuli with maximum accuracy, thus high weights are not only assigned to signal-related EEG channels (to amplify the difference) but also ‘noise-related’ EEG channels (to cancel out the discrimination-unrelated signal). The mathematical conversion from the backward to the forward model aims to isolate weights that are informative with respect to the signal and to ignore those weights that relate to the noise. The same units of measurement (µV) were obtained for the topographical maps by reversing the initial standardization step. To summarize the classification and statistical analysis results across time and sleep stages, we plot an array of matrices (later referred to as cross-generalization matrices) with colors coding classification accuracy and black dots representing significant (corrected by FDR) time points (e.g., Figure 2). All python scripts used for the analysis have been deposited on a public repository to allow the interested reader to reproduce them; DOI to access: https://doi.org/10.6084/m9.figshare.14256293 (accessed on 10 May 2021).

## 3. Results

### 3.1. Decoding of the Main Effect of VOICE Familiarity

In general, we found that a classifier trained and tested within a given sleep stage successfully discriminated between familiar and unfamiliar voices with significant (*p* < 0.01, FDR-corrected) decoding scores for NREM sleep stages N2 and N3 with an accuracy of about 60% and peaking at around 250–400 ms after stimulus onset (cf. Figure 2, diagonal). More specifically, across participants the average accuracy for the peak time window (250–400 ms) was 56.8% ± 7.9 for N2 and 55.7% ± 7.3 N3. Moreover, classifiers trained on N2 and N3 data generalized to N3 (55.4% ± 6.1) and N2 (55.2% ± 5.7), respectively, without temporal delays in the generalization patterns (Figure 2, off-diagonal). Please see the supplementary information, for more details on decoding accuracies for the peak time windows in each sleep stage (Supplementary Table S1). In the individual temporal generalization matrices for NREM stages, we observed a clear diagonal pattern with relatively short-lived (up to 100 ms) above-chance decoding, which then switches to below-chance decoding especially in N2 and N3 (Figure 2, cold colors). None of the trained NREM classifiers generalized to REM or WAKE. Likewise, classifiers trained on REM or WAKE data failed to decode voice familiarity during any other NREM sleep stage indicating that the stages are too distinct to allow for generalization of the classification. 

### 3.2. Voice Familiarity across the Brain—Topographical Patterns 

**Figure 3:**
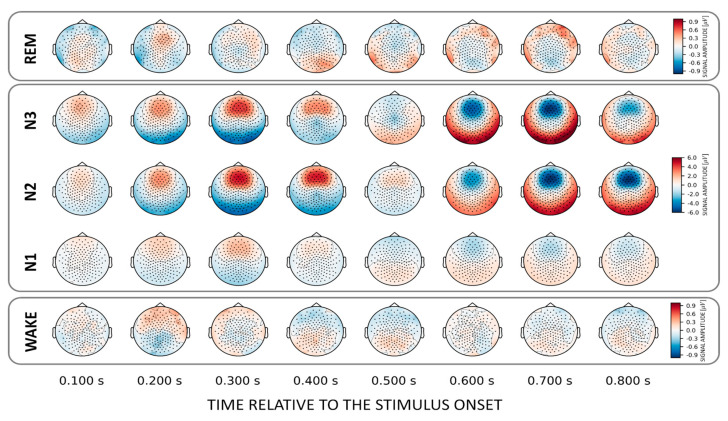
Topographical patterns obtained from the trained classifiers for the VOICE effect. Note the similar fronto–central topographical pattern in NREM stages N2 and N3. Although this pattern remains topographically stable over time, a polarity reversal can be observed around 0.6 s after stimulus onset. Note the different color scaling for WAKE and REM indicating much weaker discrimination between conditions as compared to NREM.

To localize those areas that drive the classification between familiar and unfamiliar voices we investigated (forward-transformed) weight vectors obtained from the trained classifier. We found that NREM sleep stages N2 and N3 show a common discriminatory topography around fronto–central electrodes. Interestingly, the observed topography reverses its polarity from positive to negative after about 0.6 s relative to stimulus onset (Figure 3). Note that there is also a weak pattern in the awake state over temporal electrodes around 200 ms post-stimulus onset (Figure 3, lower inset).

### 3.3. Decoding of the NAME Effect

**Figure 4:**
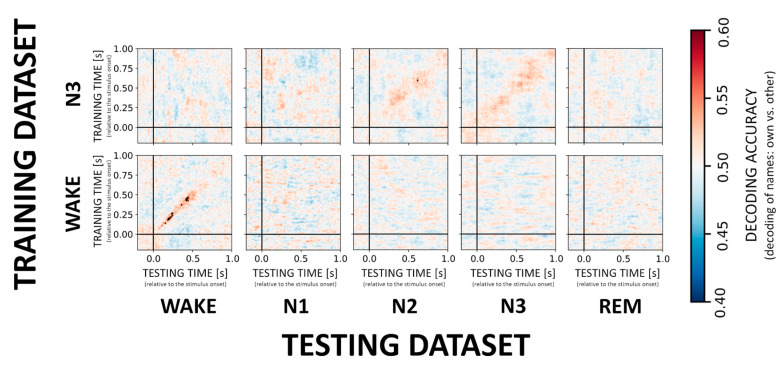
Cross-generalization matrices summarizing the decoding performance for own vs. other name processing. We here evaluate the ability of the classifier to discriminate between the NAMEs across wakefulness and sleep stages. Low and high classification accuracy is again depicted in cold and hot colors, respectively. Generalization across stages does not appear to be possible for the NAME effect with the exception of N3 generalizing to N2.

In contrast to the VOICE effect, decoding of the difference between responses to own vs. other first names (i.e., the NAME effect) appears much more difficult during sleep as compared to wake confirming earlier results by Blume and colleagues. When it comes to sleep stages, only classification in N3 showed a significant result with a classifier trained on N3 data generalizing to N2 sleep (cf. Figure 4, upper inset). Across participants average accuracy for a time window around maximal accuracy scores (250–400 ms) was 52.4% ± 2.7. Interestingly, for wake, there was a stable and significant (*p* < 0.01 FDR corrected) decoding pattern (Figure 4, inset) from 150ms onwards (on average 53.8% ± 2.0). For more details on decoding accuracies for the remaining sleep stages, please see Supplementary Table S2. Furthermore, and in contrast to VOICE decoding, we here observed only an incidental generalization during NREM and no generalization from wake to other sleep stages.

### 3.4. NAME Effect across the Brain—Topographical Patterns

**Figure 5:**
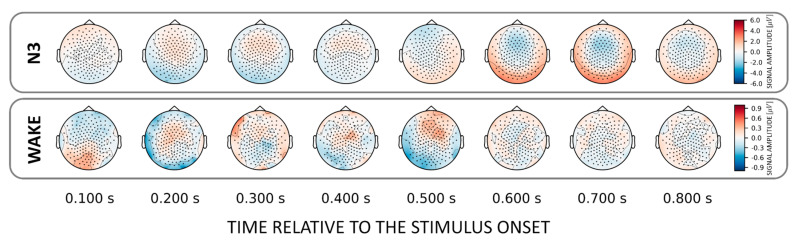
Topographical patterns obtained from the trained classifiers for the NAME effect. For N3 a topographically similar fronto-central “source” as for the corresponding decoding of voices was evident. Note the different color scalings for wake.

In general, we found that the topographical patterns for NAMEs were weaker compared to VOICE decoding. This suggests that for most states no distinct areas could be identified that allow own vs. other name stimulus categorization. Only for N3 did we again find a fronto-central discriminatory pattern that similarly to the VOICE pattern reverses its polarity after about 0.6 s post-stimulus (Figure 5, upper inset). For wakefulness, there are varying clusters starting at 150 ms and developing up to 500 ms after stimulus onset.

## 4. Discussion

In this study, we assessed whether the brain can distinguish between auditory stimuli varying in their linguistic and paralinguistic familiarity and whether the information contained in the neural responses is similar during wakefulness and different sleep stages. More specifically, we used a multivariate decoding approach to investigate the extent to which the responses to familiar vs. unfamiliar voices and own vs. other first names are similar during wakefulness and all NREM and REM sleep stages. This way, we hoped to ascertain whether the mechanisms underlying the detection of linguistic and paralinguistic salience trace back to a single neural code that is used across vigilance stages. 

By using a multivariate decoding approach, we found that the brain can indeed discriminate between familiar and unfamiliar voices during N2 and N3 sleep stages. Importantly, this effect generalized across NREM states (N2, N3). This suggests that an identical neural code is used across these states to process information as complex as the familiarity of a voice during sleep. A similar effect was also observed for N1, but after correcting for multiple comparisons, this only remained as a trend. In contrast to the first publication on this dataset [1], where differences between familiar and unfamiliar voices were evident during REM sleep, we found no significant decoding in REM. This could be due to methodological differences and specifically the use of time-frequency and ERP analyses compared to single trial decoding. Likewise, generalization was not possible from NREM to either REM or wakefulness or vice versa. We thus conclude that the neural code for familiarity discrimination inauditory stimuli is very much alike from light N2 to deep N3 sleep but quite distinct from all the other states.

Interestingly, we did not find the above chance classification in the temporal generalization matrices in N2 and N3 for the voice effect. This suggests that the underlying spatial pattern reverses its polarity [10]. Also, by investigating the topographical maps obtained for both voices (cf. Figure 3) and names (cf. Figure 5), we found a fronto–central pattern that can differentiate names and voices already 200–300 ms after stimulus-onset, but reverses its polarity around 600 ms. This means that the discriminatory pattern is topographically stable (i.e., the same EEG channels are involved), but temporally behaves like a wave with a polarity reversal starting at about 600 ms after stimulus onset. Indeed, this is in line with the finding of Blume et al. (2018) finding that a K-complex-like response follows the presentation of unfamiliar voice stimuli in all NREM sleep stages (as well as REM). According to Blume and colleagues, this could suggest increased processing and/or inhibition of salient and unfamiliar stimuli during sleep.

The present results are an extension to the original paper by Blume and colleagues [1], who found a differential response for voices during both NREM and REM. It is worth noting that decoding using machine learning as utilized here is an out-of-sample analysis, which means that the model evaluation is performed on an independent test set. This is in strict contrast to conventional approaches that use all data available for both model building and model evaluation (i.e., “in-sample” analysis). Thus, unlike conventional methods (e.g., ERP, time-frequency analyses) that allow for inference only within given datasets, a trained decoder can be used to provide direct, quantitative assessment of (dis)similarities between brain responses across different datasets.

Surprisingly, there are only very few studies in the literature in which decoding across different states of vigilance was performed and, to the best of our knowledge, this is the first study where decoding methodology is comprehensively implemented across all sleep stages (N1, N2, N3, REM and wake). For instance, Strauss et al., by investigating whether predictive-coding remains active during sleep, found significant generalization from wake to N2 but only for local (sensory) and not for global (cognitive) and attention-related processes [6]. In a similar study, there was no generalization from wake to both N2 and REM [7]. The authors concluded that information processing from wake to sleep changes substantially, with only residual processing persisting into sleep [7]. In general, these results are in line with our wake-decoding as we found no cross-generalization from wake to any sleep stage (and vice versa) for either familiar/unfamiliar voices or own/other names, which suggests that the neural code changes substantially from wake to sleep.

To conclude, we replicated results from Blume et al. [1] in the sense that the brain is able to process and distinguish acoustic stimuli during sleep. Beyond this, we show that the neural code responsible for discrimination of auditory stimuli is very much alike from light N2 to deep N3 sleep but distinct from REM or wake. Altogether, the results therefore point to the dynamic nature of the brain that become visible in the distinct vigilance states. Intriguingly, while processing of salient information continues even in non-responsive states such as sleep, the change of the vigilance state is accompanied by the use of a different “neuronal machinery” or “code”.

## Data Availability

The scripts used for data analysis have been deposited to a public data repository; DOI to access data: https://doi.org/10.6084/m9.figshare.14256293 (accessed on 10 May 2021).

## Supplementary Materials

The following are available online at https://www.mdpi.com/article/10.3390/s21103393/s1, Figure S1: Familiar vs. unfamiliar voices decoding was repeated 10 times (iteration 1–10)., Figure S2: Familiar vs. unfamiliar voices decoding was repeated 10 times (iteration 11-20). Table S1: Decoding accuracies for VOICE-mean and standard deviation across participants (N = 16) for a time window 250–400 ms after stimulus onset. Table S2: Decoding accuracies for NAME-mean and standard deviation across participants (N = 16) for a time window 250–400 ms after stimulus onset.
