# Supplementary material for: Decoding Brain Responses to Names and Voices across Different Vigilance States
Source: Sensors (Basel). 2021 May 13;21(10):3393. doi: 10.3390/s21103393 (PMC8152754; doi:10.3390/s21103393)
Supplement: Supplementary file 1 [file sensors-21-03393-s001.zip › sensors-1172768-SI.pdf]

sensors

MDPI

1. Methods – Epochs Sampling Scheme

The number of sampled epochs was set to 100 as we observed that for N1, the average number of epochs was around 100. To underline the validity of this reasoning, we here provide the mean, standard deviation and range of N1 epochs across conditions in our population:
Unfamiliar voice: 120 (+-63) 228
Familiar voice: 110 (+-58) 201
Own name: 79 (+-42) 152
Unknown name: 151 (+-79) 277

Additionally, to ensure the stability of our results with respect to the presented sampling scheme, we repeated our decoding analysis (VOICE, N2-trained) 20 times. Each run was initialized with a different random number generator. For better visibility, the obtained results are presented as two separate figures (Suppl. Figure 1 and Suppl. Figure 2). In both N2 and N3 we found that the majority of decodings yield significant results (19 out of 20 and 18 out of 20 respectively). In contrast, only 5 out of 20 results were statistically significant in N1 decoding. This result confirms the previous finding that N1 only remains as a trend.

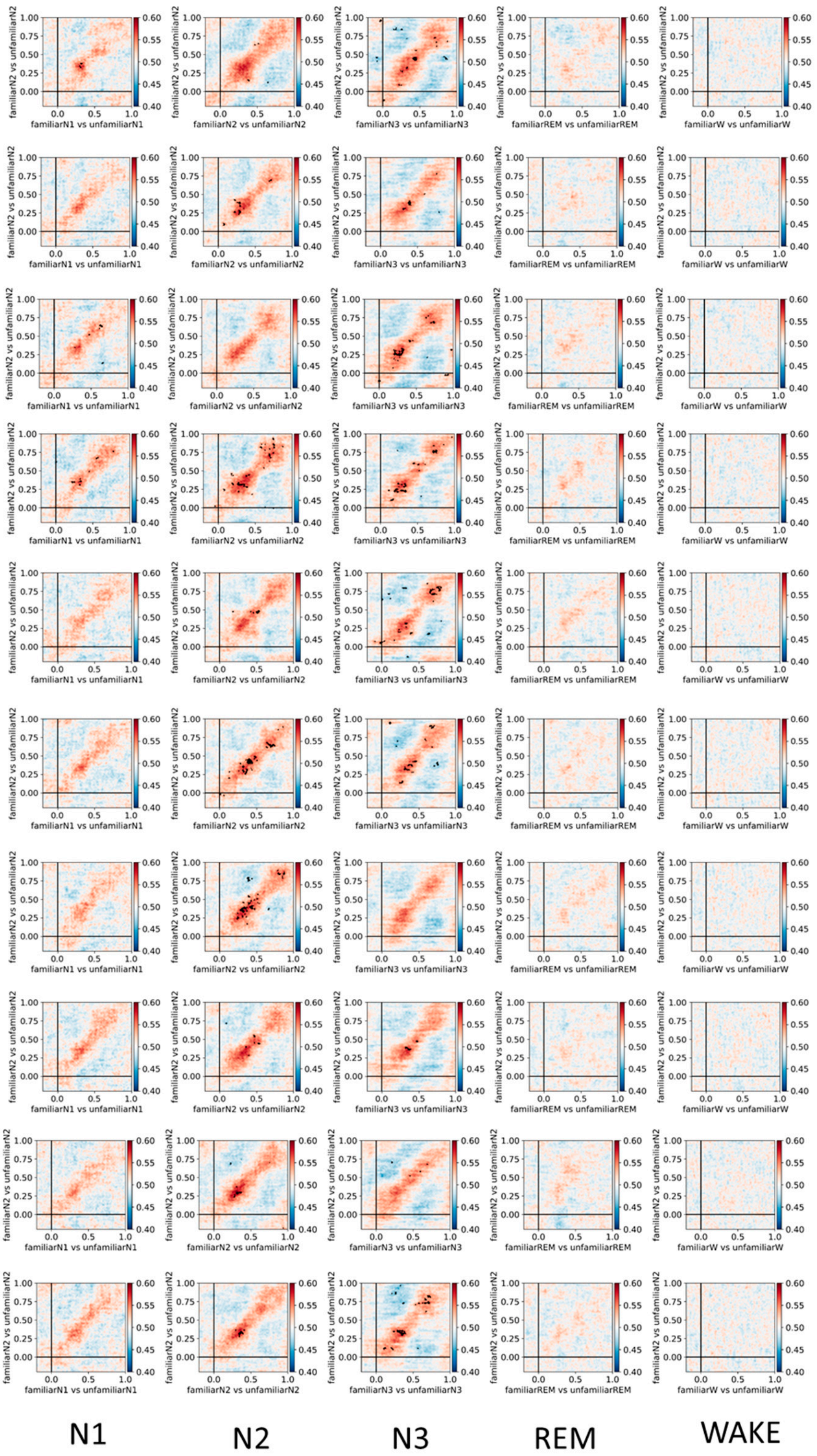

Figure 1. Familiar vs. unfamiliar voices decoding was repeated 10 times (iteration 1-10). Each row represents a classification results for a decoder trained on N2 and tested across states. Importantly, each time a random sample of 100 epochs (except N1 where all epochs were used) was taken to estimate stability of the results.

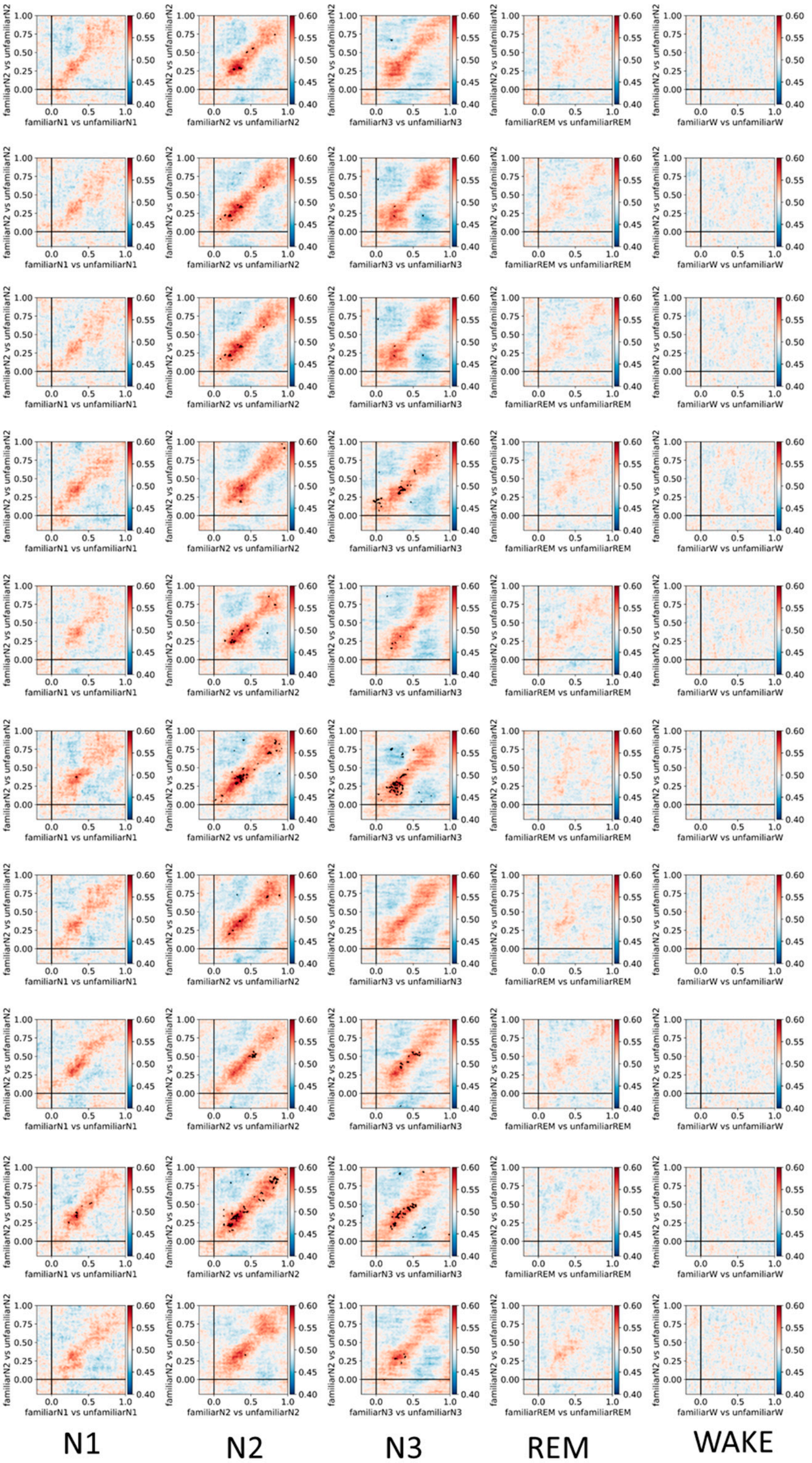

Figure 2. Familiar vs. unfamiliar voices decoding was repeated 10 times (iteration 11-20). Each row represents a classification results for a decoder trained on N2 and tested across states. Importantly, each time a random sample of 100 epochs (except N1 where all epochs were used) was taken to estimate stability of the results.

2. Results - Decoding of the Main Effect of VOICE Familiarity

Table 1. Decoding accuracies for VOICE-mean and standard deviation across participants (N=16) for a time window 250-400ms after stimulus onset.

TRAINING	REM	0.503±0.017	0.528±0.031	0.532±0.041	0.516±0.038	0.525±0.038
	N3	0.500±0.015	0.521±0.048	0.552±0.057	0.557±0.073	0.512±0.027
	N2	0.502±0.020	0.548±0.051	0.568±0.079	0.554±0.061	0.518±0.022
	N1	0.504±0.014	0.546±0.064	0.545±0.056	0.528±0.054	0.532±0.034
	Wake	0.523±0.019	0.493±0.028	0.504±0.019	0.492±0.027	0.496±0.011
	Stage	Wake	N1	N2	N3	REM
**mean±SD**				TESTING		

3. Results - Decoding of the NAME effect

Table 2. Decoding accuracies for NAME-mean and standard deviation across participants (N=16) for a time window 250-400ms after stimulus onset.

TRAINING	REM	0.501±0.014	0.516±0.036	0.510±0.011	0.504±0.016	0.511±0.018
	N3	0.496±0.015	0.512±0.022	0.524±0.027	0.524±0.034	0.504±0.014
	N2	0.503±0.013	0.519±0.030	0.515±0.037	0.511±0.022	0.511±0.021
	N1	0.502±0.019	0.510±0.036	0.515±0.035	0.509±0.017	0.506±0.020
	Wake	0.538±0.020	0.494±0.017	0.502±0.016	0.504±0.014	0.502±0.012
	stage	Wake	N1	N2	N3	REM
mean±SD				TESTING		
